# Phylogenetic analysis of the Lancinae (Gastropoda, Lymnaeidae) with a description of the U.S. federally endangered Banbury Springs lanx

**DOI:** 10.3897/zookeys.663.11320

**Published:** 2017-03-28

**Authors:** David C. Campbell, Stephanie A. Clark, Charles Lydeard

**Affiliations:** 1 Department of Natural Sciences, Gardner-Webb University, PO Box 7260, Boiling Springs, NC, 28017, USA; 2 Invertebrate Identification, 6535 N Mozart St, Chicago, IL, 60645, USA; 3 Department of Biology and Chemistry, Morehead State University, 103 Lappin Hall, Morehead, KY, 40351, USA; 4 Invertebrates, Gantz Family Collections Center, Field Museum of Natural History, 1400 S. Lake Shore Drive, Chicago, IL 60605, USA

**Keywords:** Lanx, Fisherola, Basommatophora, anatomy, molecular

## Abstract

We examined the patelliform snails of the subfamily Lancinae, endemic to northwestern North America, to test whether morphological variation correlated with genetic and anatomical differences. Molecular analyses using *cox1*, 16S, calmodulin intron, and 28S rDNA partial sequences and anatomical data supported recognition of four species in three genera. The relationships of lancines within Lymnaeidae are not yet well-resolved. The federally endangered Banbury Springs lanx is described as a new genus and species, *Idaholanx fresti*, confirming its distinctiveness and narrow endemicity.

## Introduction

The lancines are relatively large freshwater limpets (up to 20 mm in length), found from the upper Sacramento and Pit Rivers of northern California, north to the Columbia River system in the states of Idaho, Oregon, Washington and Montana in the United States and the province of British Columbia, Canada. Some freshwater limpets in related families have been shown to have high morphological variation within relatively few, widespread species (Walther et al. 2006a, b), but no previous study has analyzed the lancines in detail.

Because of their larger size and color pattern, Tryon (1870) incorrectly suspected that some lancines were mislabeled marine forms. Despite the differences, lancines were generally classified along with other freshwater limpets in Ancylinae until Pilsbry (1925) and H. B. Baker (1925) examined the anatomy and showed that they were lymnaeids. Further studies (Morrison 1955, Walter 1969) have confirmed the lymnaeid anatomy. Although several lymnaeids tend towards few whorls and wide apertures, these are the only truly patelliform members extant in the family. Within the Lancinae, three generic names have been proposed: *Lanx* Clessin, 1880, *Fisherola* Hannibal, 1912, and *Walkerola* Hannibal 1912, but whether they should be recognized as genera, subgenera, or synonyms has varied between authors. Current classification typically recognizes *Lanx* and *Fisherola* but treats *Walkerola* as a subgenus or synonym of *Lanx* (Burch & Tottenham, 1980). Nine names (plus one unpublished name cited in the literature) have been proposed for extant species (Table 1). However, there is little agreement in the literature as to whether the variation in shell shape, height, color, and anatomy between populations provide an adequate basis for recognizing all of these taxa (Morrison 1955).

**Table 1.** Nominal Recent species names in Lancinae.

Species name	Type locality	Assignment in present study
*Ancylus altus* Tryon, 1865	Klamath River	*Lanx alta* (Tryon, 1865)
*Ancylus crassus* Haldeman, 1844	Columbia drainage	*Fisherola nuttallii* (Haldeman, 1841)
*Lanx hannai* Walker, 1925	upper Sacramento River	*Lanx patelloides* (Lea, 1856)
Lanx (Walkerola) klamathensis Hannibal, 1912	Klamath River	*Lanx alta* (Tryon, 1865)
*Ancylus kootaniensis* Baird, 1863 [*kootenaiensis* is an invalid emendation]	Kootenai River (restricted by Morrison 1955)	probably *Fisherola nuttallii* (Haldeman, 1841) but not directly sampled
*Fisherola lancides* Hannibal, 1912	Snake River	*Fisherola nuttallii* (Haldeman, 1841)
*Ancylus newberryi* Lea, 1858	upper Sacramento (correction by Pilsbry 1925)	*Lanx patelloides* (Lea, 1856)
Ancylus (Velletea) nuttallii Haldeman, 1841	Columbia drainage	*Fisherola nuttallii* (Haldeman,1841)
*Ancylus patelloides* Lea, 1856	upper Sacramento River	*Lanx patelloides* (Lea, 1856)
*Ancylus praeclarus* Stimpson ms. cited in Lea, 1867	unstated	not validly proposed; Lea stated that *newberryi* differs from it in several ways but never directly said anything about *praeclarus*
*Ancylus subrotundatus* Tryon, 1865	Umpqua River	*Lanx alta* (Tryon, 1865)

Of particular importance are the questions relating to the status of the Banbury Springs lanx. Banbury Springs lanx was discovered by Terry Frest in 1988 and thought to be a new, undescribed species within the genus *Lanx*. It is listed as federally endangered in the United States (U.S. Fish and Wildlife Service 1992). Although the small size and different shape distinguish it from other lancines, normal *Fisherola* occur nearby in the Snake River, raising the possibility that it is just a local ecomorph. However, no populations of *Fisherola* are known from any other springs (U.S. Fish and Wildlife Service 2006).

The primary objective of this study was to determine the taxonomic status of the United States federally endangered Banbury Springs lanx. We describe it as a new genus and species based on molecular and anatomical data. Secondly, we examine the phylogenetic relationships of the Lancinae using mitochondrial and nuclear gene regions.

## Materials and methods

We sampled populations from throughout the geographic range of *Lanx* and *Fisherola*, emphasizing morphologically or geographically distinct populations (Table 2). A few additional lymnaeids were sampled as outgroups. Specimens were preserved in ethanol in the field. Dissections were carried out using a stereomicroscope fitted with a camera lucida. Typically at least two specimens per population were dissected; in some cases only one specimen was available. DNA extraction used digestion in CTAB overnight at 37°C, followed by chloroform-isoamyl alcohol separation, isopropanol precipitation, and washing with 70% ethanol before drying and dissolving in TE (Campbell et al. 2005). PCR amplification was often difficult, so several genes were attempted in an effort to find genes with suitable variation that amplified consistently. ITS failed to amplify. 16S (using the primers from Krebs et al. 2003) amplified for few populations. *Cox1* (using primers LCO1490 from Folmer et al. 1994 and the external primer from Carpenter and Wheeler 1999) amplified for several but not all samples. The best amplification was obtained for 28S (primers 2/3F and 6R from Park and Ó Foighil 2000) and calmodulin intron (primers from Schilthuizen et al. 1999 and new primers ATGAAGTGGATGCTGAYGG and ATTCTGGGAARTCTATYG). However, as observed for other gastropods (Simpson et al. 2005), multiple highly divergent calmodulin intron alleles were obtained, suggesting that multiple copies of calmodulin exist in basommatophorans. The sequence length variation was sufficient to make selection of a single copy straightforward using gel extraction (QIAquick gel extraction kit, Qiagen). The band of about 420 bp (including primers) was selected because it consistently amplified strongly. Because the key variable region in 18S is in the first part of the gene, we used the 1F-4R primers (Giribet et al. 1996) to amplify that portion of the gene. PCR cycles used were 95°C, 3 min; 5 cycles at 92°C for 30 sec, 40°C for 30 sec, 65°C for 2 min; and 40 cycles with at 92°C for 30 sec, x°C for 30 sec, 65°C for 2 min, where x is about 2°C below the lower primer annealing temperature; finishing with 10 min at 72°C before cooling to 4°C. In some cases with weak amplification, nested PCR for calmodulin intron using the Schilthuizen et al. (1999) primers followed by the new primers was used. PCR products were purified using DyeEx 2.0 kits (Qiagen). Sequencing used ABI BigDye 3.1 with cycle sequencing reactions of 4 minutes at 96°C, followed by 40 cycles with 15 sec at 96°C, 15 sec at about 2°C below the lower primer annealing temperature, and 4 min at 65°C, followed by 10 min at 72°C before cooling to 4°C. Sequences were aligned in BioEdit 7.0.5.3 (Hall 1999). Preliminary alignments made use of CLUSTAL W (Larkin et al. 2007), followed by manual editing to eliminate unnecessary gaps, inconsistent alignment of identical sequences, and other problems. Outgroups were selected based on the availability of 28S sequence data and at least one of the other included genes. To obtain more complete genetic coverage, three outgroups (*Carinifex* sp., *Polyrhytis emarginata* *s.l.*, and *Galba modicella* *s.l.*) combined sequences from more than one nominal species, but the species in question are closely related and have sometimes been synonymized.

**Table 2.** Populations sequenced. Species names under “Morphospecies” were assigned based on shell form. Designation is the name assigned based on the present results and used in the trees. A single individual from the Rogue system yielded two distinct calmodulin intron sequences and unique sequences for 28S and *cox1*.

Designation	Morphospecies	Locality	Drainage	Accession number
*Idaholanx fresti*	Banbury lanx	Banbury Springs, Idaho	Snake	calmodulin HM230326, 28S HM230308, *cox1* HM230356, 16S KT267273
*Idaholanx fresti*	Banbury lanx	Box Canyon Springs, Idaho	Snake	calmodulin HM230327, 28S HM230309, *cox1* HM230357, 16S KT267273
*Idaholanx fresti*	Banbury lanx	Briggs Spring, Idaho	Snake	28S HM230310
*Idaholanx fresti*	Banbury lanx	Thousand Springs, Idaho	Snake	calmodulin HM230328, 28S HM230311
*Fisherola nuttallii*	*F. lancides*	off Bancroft Springs, Snake River, Idaho	Snake	calmodulin HM230330, 28S HM230315, *cox1* HM230359, 16S HM230355
*Fisherola nuttallii*	*F. nuttallii*	Deschutes River, RM 6.3, Oregon	Columbia	calmodulin HM230329, 28S HM230314, 16S KT267274
*Fisherola nuttallii*	*F. nuttallii*	Owyhee River, Whistling Bird Rapids, Oregon	Snake	calmodulin HM230331, 18S HM230306, 28S HM230316, *cox1* HM230360
*Lanx alta*	*L. alta*	Klamath River at Collier Rest Area, California	Klamath	calmodulin HM230336, 18S HM230307
*Lanx alta*	*L. klamathensis*	Barclay Spring, Hagelstein Park, Upper Klamath Lake, Oregon	Klamath	calmodulin HM230335, 28S HM230319
*Lanx alta*	*L. klamathensis*	Link River at Hwy bridge, Klamath Falls, Oregon	Klamath	calmodulin HM230337
*Lanx alta*	*L.* species	Smith River National Recreation Area, California	Smith	calmodulin HM230341, 28S HM230321
*Lanx alta*	*L.* species	Smith River National Recreation Area, California	Smith	calmodulin HM230342
*Lanx alta*	*L.* species cf. *L. alta*	Rogue River at Gold Nugget Recreation area (BLM), Oregon	Rogue	calmodulin HM230338, HM230340 (identical sequence from two specimens)
*Lanx alta*	*L.* species cf. *L. alta*	Rogue River at Gold Nugget Recreation area (BLM), Oregon	Rogue	calmodulin HM230339, 28S HM230320, *cox1* HM230362
*Lanx alta*	*L. subrotundata*	Amacher City Park, Roseburg, Umpqua River, Oregon	Umpqua	calmodulin HM230334, 28S HM230318, *cox1* HM230361
*Lanx patelloides*	*L. hannai*	McCloud River S. of Ah-Di-Na Camp Ground, California	Sacramento	calmodulin HM230346, 28S HM230322, *cox1* HM230363
*Lanx patelloides*	*L. patelloides*	Battle Creek, Sacramento River, California	Sacramento	calmodulin HM230343
*Lanx patelloides*	*L. patelloides*	Pit River at CA Hwy 299 bridge, California	Sacramento	calmodulin HM230347
*Lanx patelloides*	*L. patelloides*	Sucker Springs lower spring channel, California	Pit	calmodulin HM230348, 28S HM230323
*Lanx patelloides*	*L.* species	Lava Creek Lodge, Eastman Lake, Fall River, California	Pit	calmodulin HM230344, HM230349(long), 16S KT267276
*Lanx patelloides*	*L.* species	Lost Creek source spring	Pit	calmodulin HM230345

DNA data were analyzed in PAUP* 4.0a152 (Swofford 1998), TNT (Goloboff et al. 2008) and MrBayes3.2 (Ronquist et al. 2011). Duplicate sequences were eliminated from the phylogenetic analyses. Partition-homogeneity tests (P_ILD_ of Dowton and Austin 2002) were run in PAUP*4.0a152 with 100 replicates of 10 random addition replicates each. This test is sensitive to other factors, such as partition size and evolutionary model, besides data compatibility (Dowton and Austin 2002), but may provide a rough idea of agreement between data sets. Despite the problems of the ILD type of tests, no better alternative has gained wide acceptance. The test requires data for each included taxon and partition, so pairwise comparisons were made between all genes. The only significantly incompatible gene was 16S data, so it was analyzed separately, but the others were concatenated. Indels were coded as missing data. Parsimony analyses in PAUP* used 500 replicates of TBR swapping, with random taxon addition sequence and holding 10 trees at each addition step. Parsimony bootstrapping used 500 replicates, each replicate being a random-addition heuristic search with 10 random replicates. MrModeltest 2.2 (Nylander 2004) was used to select a maximum likelihood model for the nucleic acid sequences that was then input into MrBayes. Bayesian analyses used 2,000,000 generations and 8 chains, with revmat, shape, pinvar, and statefreq unlinked, and the concatenated sequence had the genes identified as partitions. Duplicate sequences were excluded.

### Abbreviations

**FMNH** Field Museum of Natural History, Chicago, Illinois, U.S.A.

**SAC** Invertebrate Identification’s invertebrate reference collection, Chicago, Illinois, U.S.A.

**DCS** Deixis Consultants mollusc reference collection, Seattle, Washington, U.S.A.

**Table 3.** Outgroup sequences analyzed. Source gives locality for new specimens and literature citation for published sequences. * indicates newly generated sequences.

Taxon	Gene	Accessions	Sources
*Acroloxus lacustris* (Linnaeus, 1758)	16S	AY577462	Jorgensen et al. 2004
*Acroloxus lacustris* (Linnaeus, 1758)	28S	DQ328296	Walther et al. 2006b
*Acroloxus lacustris* (Linnaeus, 1758)	*cox1*	DQ328271	Walther et al. 2006b
*Ancylus fluviatilis* Müller, 1774	16S	AY577466	Jorgensen et al. 2004
*Ancylus fluviatilis* Müller, 1774	28S	DQ328295	Walther et al. 2006b
*Ancylus fluviatilis* Müller, 1774	*cox1*	DQ328270	Walther et al. 2006b
*Austropeplea tomentosa* (L. Pfeiffer, 1855)	16S	EU556238	Puslednik et al. 2009
*Austropeplea tomentosa* (L. Pfeiffer, 1855)	28S	HQ156217	Holznagel et al. 2010
*Austropeplea tomentosa* (L. Pfeiffer, 1855)	*cox1*	AY227365	Remigio and Hebert 2003
*Carinifex newberryi* (Lea, 1858)	28S	*HM230312	Lava Creek, 1st spring pool N. of Hanna Boathouse, CA
*Carinifex ponsonbyi* Smith, 1876	16S	*HM230354	Hagelstein Park, mid channel E. side center, Klamath River, OR
*Carinifex ponsonbyi* Smith, 1876	*cox1*	*HM230358	Hagelstein Park, mid channel E. side center, Klamath River, OR
*Dilatata dilatata* (Gould, 1841)	28S	*HM230313	Sipsey River near Benevola, Greene Co. AL
*Dilatata dilatata* (Gould, 1841)	*cox1*	EF012173	Albrecht et al. 2007
*Galba modicella* (Say, 1825)	*cox1*	KM612000	Dewaard et al. 2015
*Galba obrussa* (Say, 1825)	16S	AF485658	Remigio 2002
*Galba obrussa* (Say, 1825)	28S	*HM230317	Sipsey River near Benevola, Greene Co. AL
*Galba obrussa* (Say, 1825)	cam	*HM230332	Sipsey River near Benevola, Greene Co. AL
*Lymnaea stagnalis* (Linnaeus, 1758)	16S	AF485661	Remigio 2002
*Lymnaea stagnalis* (Linnaeus, 1758)	28S	AY427490	Vonnemann et al. 2005
*Lymnaea stagnalis* (Linnaeus, 1758)	*cox1*	KT831385	Gordy et al. 2016
*Orientogalba ollula* (Gould, 1859)	16S	U82067	Remigio and Blair 1997
*Orientogalba ollula* (Gould, 1859)	28S	AY465065	Jung et al., unpublished
*Orientogalba ollula* (Gould, 1859)	*cox1*	KC135900	Park et al. 2012
*Physa acuta* (Draparnaud, 1805)	16S	JQ390525	Nolan et al. 2014
*Physa acuta* (Draparnaud, 1805)	28S	DQ256738	Holznagel et al. 2010
*Physa acuta* (Draparnaud, 1805)	*cox1*	JQ390525	Nolan et al. 2014
*Planorbella trivolvis* (Say, 1817)	16S	AY030234	DeJong et al. 2001
*Planorbella trivolvis* (Say, 1817)	28S	AF435688	Morgan et al. 2002
*Planorbella trivolvis* (Say, 1817)	*cox1*	KM612028	Dewaard et al. 2015
*Polyrhytis emarginata* (Say, 1821)	28S	DQ328299	Walther et al. 2006b
*Polyrhytis elodes* (Say, 1821)	16S	AF485652	Remigio 2002
*Polyrhytis exilis* (Lea, 1834)	*cox1*	*HM230364	Ditch along the Stump Lake access road, Jersey Co., IL
*Radix auricularia* (Linnaeus, 1758)	16S	JN794284	von Oheimb et al. 2011
*Radix auricularia* (Linnaeus, 1758)	28S	AY465067	Jung et al., unpublished
*Radix auricularia* (Linnaeus, 1758)	*cox1*	KP242340	Patel et al. 2015
*Radix balthica* (Linnaeus, 1758)	16S	HQ330989	Feldmeyer et al. 2010
*Radix balthica* (Linnaeus, 1758)	28S	EF417136	Sonnenberg et al. 2007
*Radix balthica* (Linnaeus, 1758)	*cox1*	KP098541	Feldmeyer et al. 2015

## Results

Amplification of 28S and calmodulin intron were most successful, but representatives of each species (as recognized herein) also amplified for *cox1*. Within Lancinae, interspecies and intergenus percent variation was lowest for 28S and highest for *cox1*. However, the calmodulin intron sequence for lancines was more divergent from *Galba obrussa* than the maximum variation between lymnaeids for *cox1* (26-30% versus 22%) (Table 4). Calmodulin sequences for planorbids generated in ongoing study on *Vorticifex* were apparently homologous based on the beginning and end of the intron sequence, but the middle of the intron was too divergent in sequence and length to obtain a meaningful alignment between the planorbids and lymnaeids. One calmodulin intron paralog of significantly different length was sequenced, but no homology with the chosen paralog was evident (GenBank accession number HM230349).

**Table 4.** Range of percent differences in DNA sequence (raw data, gaps treated as missing).

Gene	Lymnaeidae	lancine genera	Lanx species	lancine intraspecies
28S	up to 7.6%	1.2–2.8%	0.79–1.2%	0.00–0.40%
CAM intron	up to 30.1%	4.8–8.0%	1.3–2.6%	0.00–1.87%
*cox1*	up to 21.1%	12.9–21.1%	7.9–8.6%	0.15–1.0%
16S	up to 21.3%	12.8–16.6	no data	0.00–2.5%

**Figure 1.**
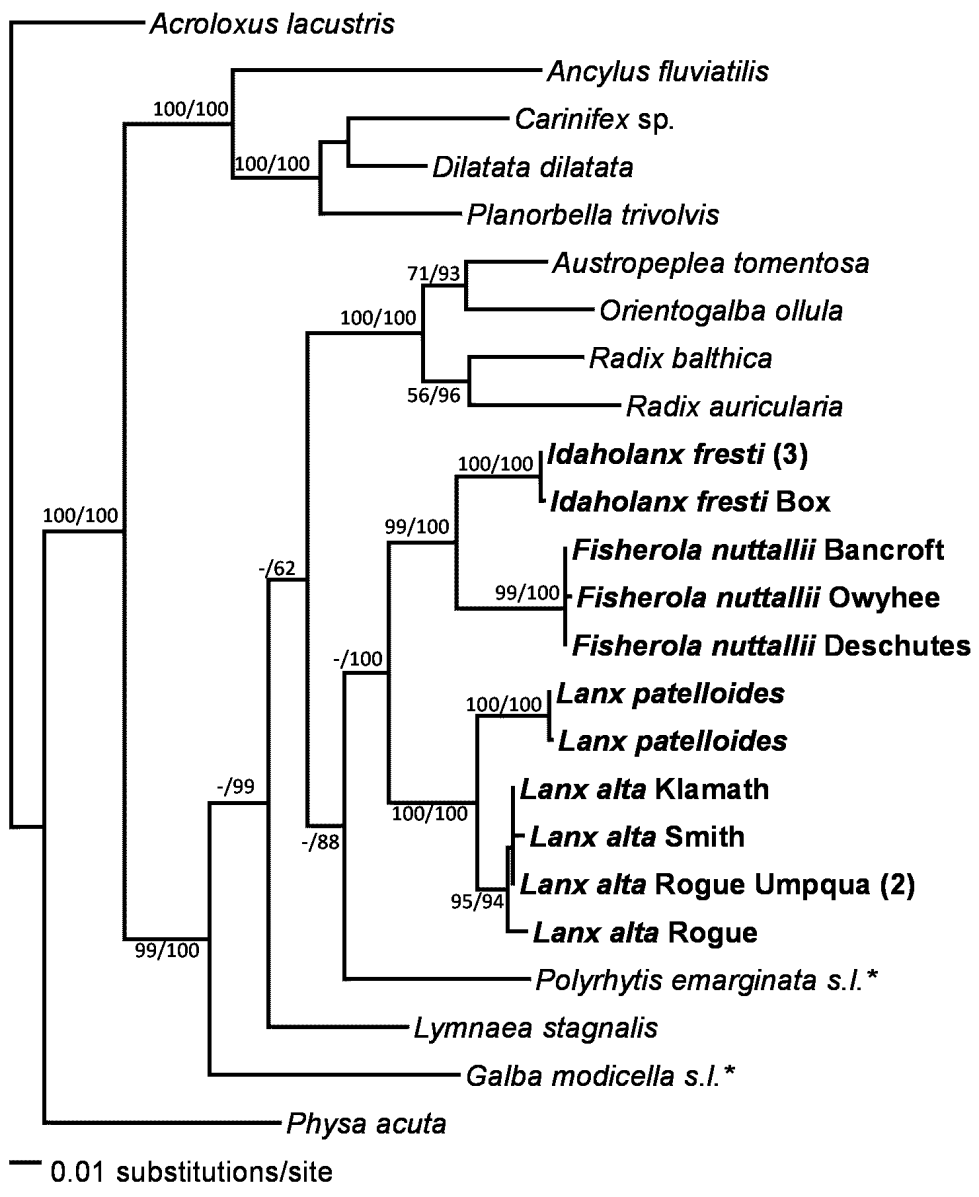
Phylogram of the Bayesian majority-rule consensus tree for 28S, *cox1*, and calmodulin intron sequence data. Numbers on branches are bootstrap percentages before the slash, then Bayesian posterior probabilities. - indicates a value under 50% or 0.5 when the other method gave higher values. Taxon names in bold are lancines; starred taxa are Acellinae.

**Figure 2.**
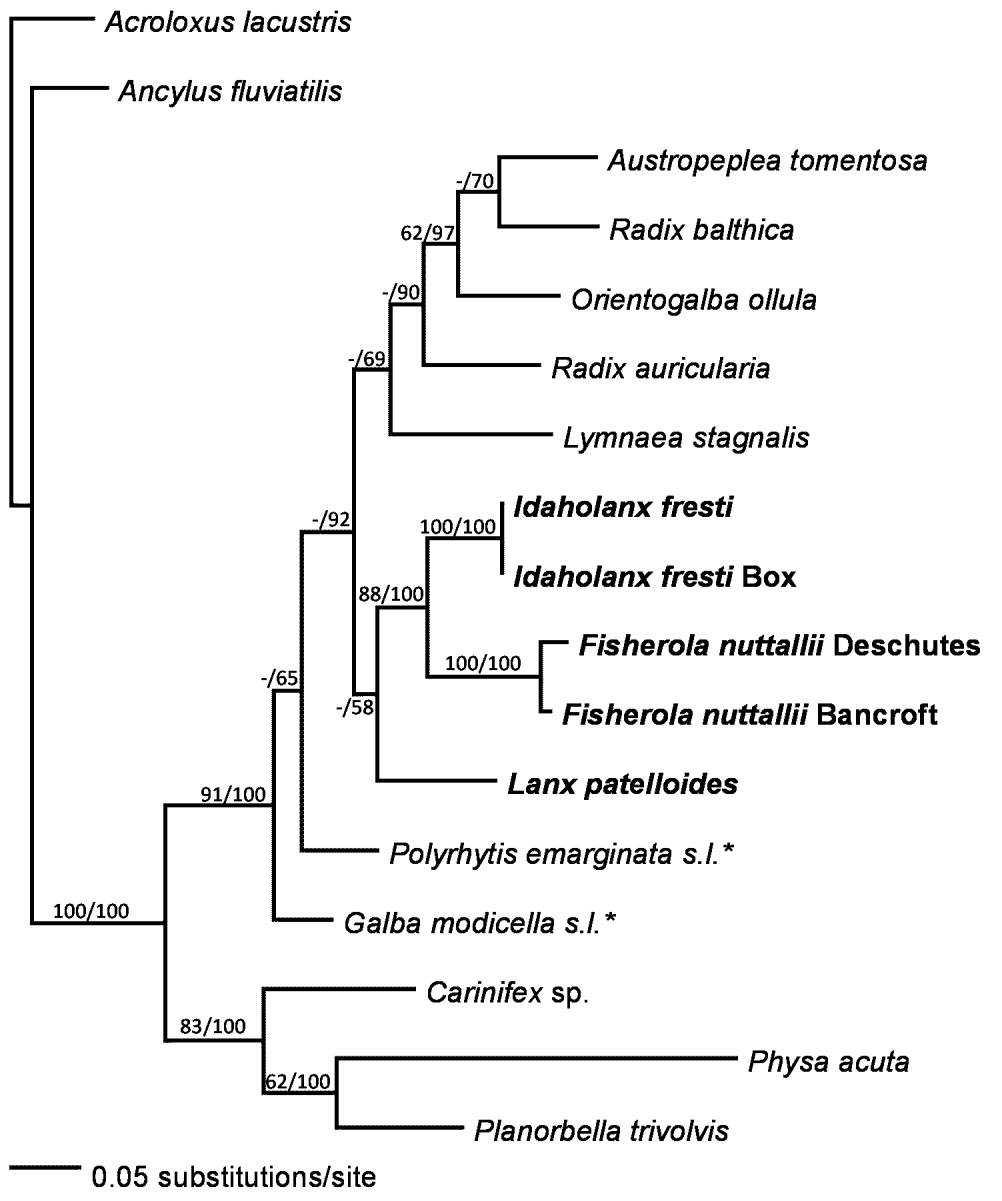
Phylogram of the Bayesian majority-rule consensus tree for 16S sequence data. Numbers on branches are bootstrap percentages before the slash, then Bayesian posterior probabilities. - indicates a value under 50% or 0.5 when the other method gave higher values. Taxon names in bold are lancines; starred taxa are Acellinae.

Several populations yielded identical or nearly identical sequences. These are enumerated in Table 2. No indels were found in *cox1* within the sampled species, though other Hygrophila do have insertions (pers. obs.). 28S, 16S, and calmodulin intron all had several small indels. MrModeltest (Nylander 2004) favored a HKY model for calmodulin intron and GTR+I+G for 28S, 16S, and *cox1*. Figures 1–2 show the results of phylogenetic analyses.

Parameters for the trees from these analyses are in Table 5. All Bayesian analyses had a final average standard deviation of split frequencies below 0.6%. Roughly 70% bootstrap support or 95% Bayesian posterior probability are thought to reflect significant support, though these empirical estimates are affected by several data and tree characteristics.

As 18S typically shows little resolution at the species level, it was only sequenced for two species from different lancine genera, and those sequences were identical. Table 6 gives the E10-1 variable region for lymnaeids (present results and published data). The sequences are sufficiently variable to make alignment uncertain. Parsimony analyses using different alignments gave substantially different phylogenetic patterns, so we did not use them. The alignment in the table is to facilitate comparison and may not reflect actual homology. However, several groups of species have closely similar or identical sequences, supporting a close relationship within these groups.

**Table 5.** Tree statistics.

Gene	Parsimony		Bayesian		
	# trees	length	burnin	maximum ln likelihood	mean ln likelihood
28S, CAM intron, and *cox1*	18	1670	165000	-9578.885	-9602.83
16S	2	719	65000	-3414.11	-3427.56

**Table 6.** Hypervariable portion of the E10 region of 18S genes for lymnaeids. * indicates newly generated data. The alignment is meant to facilitate comparison between the different species. Differences between the more divergent sequences are too great for confident homologizing.

Species	Accession number	Sequence
*Aenigmomphiscola europaea, A. kazakhstanica, Lymnaea stagnalis, Omphiscola glabra, Stagnicola palustris*	AY577484, FR797819-FR797829, JN614363, JN614364, HQ659966, JN614368, JN614367	CCGCG------TGC-GG--GGCGACTCGT-GCGCGGCG
*Fisherola nuttallii*	HM230306*	CCGT-CGC-GCGGGGCGTCAAACCCTCGCCG-GCGGCG
*Galba cousini*	FN598151, JN614345, JN614344	CCGT-------CGCGGCGCAAGCCGAG-----GCGGCG
*Galba cubensis*	Z83831, JN614326-JN614331, JN614334	CCGTGTCGTGCCGCGGTGCAAGCCGTGGTCGCGCGGCG
*Galba humilis*	FN182190	CCGT-------CGCGGCGCAGGCCGAG-----GCGGCG
*Galba schirazensis*	FR772291, JN614335-JN614343	CCGGC----CATTCATTCACTTGCGTGG----TCGGCG
*Galba truncatula*	Y09019, Z73985, EU152270, EU728668, HQ659965, JN614346-JN614354, FR797815, FR797816	CCGT-------CCT-TTC----GCGAGG----GCGGTG
*Galba viator*	AF239912	CCGTGTGCCTCCGTGGTGCAAGCCGTGGTCGCGCGGCG
*Galba viator*	AM412222, AY057088, AY057089, JN614332, JN614333	CCGTGTGCCTCCGCGGTGCAAGCCGTGGTCGCGCGGCG
*Lanx alta*	HM230307*	CCGT-CGC-GCGGGGCGTCAAACCCTCGCCG-GCGGCG
*Lymnaea stagnalis*	EF489345	CCG------------------------------CGGCG
*Lymnaea stagnalis, Omphiscola glabra, Stagnicola palustris*	Y09018, Z73984, AY427525,Y09015, Z73982, JN614365, JN614366, Y09016, Z73983	CCGCG------TGCCGG--GGCGACTCGT-GCGCGGCG
*Pectinidens diaphana*	EU241865, JF909497, JN614361, JN614362	CCGC-------CGC-GG--CTCGCGCCGT-G-GCGGCG
*Pseudosuccinea columella*	FN598152, JN614358-JN614360	CCGT-------CGGTCC--CGCGAGGGGCCG-GCGGTG
*Pseudosuccinea columella*	EU241866	CCGTT------CGGTCC--CGCGAGGGGCCG-GCGGTG
*Radix auricularia, Radix peregra*	Z73980, Y09017, Z73981, FR797817, FR797818, JN614356, JN614357	CCGCG------TGCTC---TTCGCGGGGT-GCGCGGTG
*Radix natalensis*	AF192272, EU152269	CCGCG------TGCTC---CTCACGGGGT-GCGCGGTG
*Radix natalensis*	AF192273	CCGCG------TGCTC---CTCACGGGGT-GCGTGGTG
*Radix natalensis*	AF192274	CCGCG------TGCTC---CTCCCGGGGT-GCGCGGTG
*Radix natalensis*	JN614355	CCGCG------TGCTC---CTCGCGGGGT-GCGCGGTG

Sources: Bargues and Mas-Coma 1997 (Z73980-5); Bargues et al. 1997 (Z83831); Bargues et al. 2007 (AM412222); Bargues et al. 2009 (FN182190); Bargues et al. 2011a (FR772291); Bargues et al. 2011b (FN598151-2); Bargues et al. 2012 (JF909497); Correa et al. 2011 (JN614326-68); Dayrat et al. 2011 (HQ659965-6); Duffy et al. 2009 (AF239912, AY057088-9, EU241865-6, EU728668); Jorgensen et al. 2004 (AY577484); Klamath River at Collier Rest Area, California (HM230307*); Klussmann-Kolb et al. 2008 (EF489345) (Note: their analyses excluded variable regions, so perhaps the region was excised from the published sequence rather than originally absent); Marquez, unpublished (Y09015-9); Owyhee River, Whistling Bird Rapids, Oregon (HM230306*); Stothard et al. 2000 (AF192272-4); Vinarski et al. 2011 (FR797815-29) Vonnemann et al. 2005 (AY427525); Walker et al. 2008 (EU152269, EU152270). Only the E10 region is considered above, so there may be differences in other parts of the sequence for ones that are grouped in the table.

## Discussion

In agreement with the anatomical data, molecular data give strong support for placing Lancinae in Lymnaeidae, which favors treating lancines as a subfamily rather than as a separate family. The relationships of lancines to other lymnaeids are not yet well-resolved. Anatomy (Walter 1969) supports an affinity between Lancinae and the predominantly New World “advanced stagnicoline” group (subfamily Acellinae). Amphipepleinae (*Radix, Austropeplea*, and *Orientogalba*) was consistently supported as monophyletic, but the relationships between Amphipepleinae, Lancinae, and the remaining lymnaeids were not well-resolved, probably a function of the limited number of taxa. Sampling of additional lymnaeids, as well as additional genetic data (especially 28S) should greatly improve resolution of the relationships in this diverse and important but taxonomically problematic family.

The Lancinae appear supported as a monophyletic group, relatively divergent from other lymnaeids. Most of the analyses, the 18S sequence similarity, and several morphological features all support Lancinae. Morphological synapomorphies include the fully patelliform shell, shape of the penial complex and C-shaped to circular columellar muscle (Baker 1925, this work). Patelliform lymnaeids evolved convergently multiple times in the Miocene Paratethys lakes of southeastern Europe (Harzhauser and Mandic 2008), so the molecular data provides a useful test of the morphological similarities. However, the monophyly of Lancinae received low bootstrap support and, in the 16S analysis, low Bayesian posterior probabilities. Within the Lancinae, the present analyses had *Idaholanx* more closely related to *Fisherola* than to *Lanx*. Some single-gene analyses (not shown) had other patterns of intergeneric relationships in Lancinae. The weak resolution may reflect the limited number of available outgroups with 28S data. Additionally, variation in the *cox1* gene may be approaching saturation within Lancinae, as the maximum percent difference between lancines, the maximum difference between any two lymnaeids, and the differences between lymnaeids and other basommatophorans were all about 20%. As a result, convergent effects of multiple mutations in the variable sites probably obscure higher-level relationships in this data set. MacNeil (1939) reported Cretaceous lancines, so the subfamily has had enough time to develop significant genetic variation.

The genetic data consistently support recognition of three major groups within Lancinae. Two correspond to the presently recognized genera *Lanx* and *Fisherola*, while the third includes only the Banbury lanx. These results suggest that the Banbury lanx deserves recognition as a distinct genus and species (see description below). Each lancine genus was strongly supported as monophyletic. Genetic variation within *Fisherola* and *Idaholanx* was minimal. Within *Lanx*, there was one clear division and one ambiguous division between populations. The Sacramento-Pit system populations of *Lanx* (*L. patelloides*) consistently differed from those from farther west and north. These western and northern *Lanx* populations include *L. alta* in the Klamath and Umpqua systems and genetically more variable populations from the Smith and Rogue River systems. The difference between the Smith and Rogue forms and standard *L. alta* was less than the difference between *L. alta* and *L. patelloides* (in the case of 28S, only a few bases) but greater than the variation within other drainages. One specimen from the Rogue River system had both the standard *L. alta* allele and the Smith River allele for calmodulin intron, and the two calmodulin intron alleles obtained for Smith River specimens appear paraphyletic relative to the standard *L. alta* allele. The variation within the Rogue and Smith systems therefore appears infraspecific, and the populations are assigned to *L. alta*. However, the genetic variation may be evolutionarily significant for the conservation of this species. H. B. Baker (1925) and Morrison (1955) noted that the Rogue River population did not exactly match described species from other drainages. *Lanx alta*, as defined herein, is very plastic in shell shape, so this may not be significant.

The relatively high genetic differences between lancine species contrasts with many other lymnaeids. The present results suggest that only one lancine species is present in each river system, with the exception of *Idaholanx fresti* in a few springs and *Fisherola nuttallii* in the main rivers, both in the Columbia-Snake system. The recognition of only two species in *Lanx* contrasts with most previous classifications. In particular, the widely recognized *L. subrotunda* and *L. klamathensis* are synonymized herein with *L. alta*. Previous tentative synonymization of *L. hannai* with *L. patelloides* and *F. lancides* with *F. nuttallii* are also supported (Morrison 1955, Burch 1982). Although specific populations assigned to *F. kootaniensis* and *L. newberryi* were not sampled in this study, the observed lack of variation within river systems supports previous synonymization with *F. nuttallii* and *L. patelloides*, respectively (Pilsbry 1925). Pilsbry (1925) also pointed out that *F. crassus* is an objective synonym of *F. nuttallii*, Haldeman having apparently renamed the same specimen. These synonymies suggest that lancines are relatively variable in shell shape and color pattern, as suspected by Morrison (1955). Similar results from Walther et al. (2006a, b) for the ancylids *Ferrissia* and *Laevapex* suggests that limpet-shaped Hygrophila have been taxonomically oversplit due to ecomorphic variation. Effects of environmental parameters correlate with shell shape in limpets (Basch 1963, McMahon and Whitehead 1987, Tanaka et al. 2002), and there is also extensive unexplained variation within populations (McMahon 2004). Additionally, limpet shape may be affected by the available substrate (Ridgway et al. 1999). Albrecht et al. (2004) discuss several factors potentially influencing shell shape in freshwater limpets and suggest that waves or currents and predators are the most likely selective pressures. Denny (2000) found that marine intertidal limpets are not optimized to resist wave-produced forces, presumably because the grasping force of a stationary marine limpet typically greatly exceeds observed wave forces. However, the smaller size and thin shells of freshwater limpets and the different environmental parameters for a stream with continual flow versus unpredictably directed waves during tide changes may result in different environmental pressures. Evolutionary pressures and convergent evolution relating to the limpet shape are reviewed in Vermeij (2016), including discussion of the lymnaeids.

The potential for self-fertilization in Hygrophila may account for high genetic divergence. Self-fertilization varies from rare to common in different species (Njiouku et al. 1993, Dillon et al. 2005, Puurtinen et al. 2007). The ultimate population bottleneck of a single individual would produce extreme founder effects and genetic drift, while also producing a genetically uniform founding population, thus accounting for high divergence between taxa and low variation within. Bolotov et al. (2016) found evidence for high divergence due to founder effect in the postglacial invasion of Iceland by lymnaeids. Although the long geologic history of lancines would allow for plenty of time to accumulate changes, if the modern genera diverged fairly early, the lancines are unusually divergent in *cox1* protein sequence relative to the other lymnaeids, suggesting additional factors at work. Variation between populations within a river system was quite low. The largest difference between any two alleles within a river system was 9 to 10 bases between calmodulin intron alleles in the Smith and Rogue River populations. Outside of those, there was one individual of *L. alta* from the Klamath River with a single deletion of 6 bases in the calmodulin intron.

The low species diversity of lancines (four species from the entire Pacific Northwest region) contrasts with freshwater caenogastropods such as *Juga* and *Fluminicola* in the same river systems, which show high local endemicity within drainages (Hershler et al. 2007, Campbell et al. 2016). The habitat preferences of lancines resemble those of the associated caenogastropods, primarily in cool, flowing, well-oxygenated water, often in springs or spring-influenced areas. The potential for a single hermaphroditic individual to found a new population facilitates dispersal in Hygrophila, in contrast to the gonochoristic caenogastropods. However, unlike many lymnaeids, lancines have a poorly developed lung and are not known to survive out of water for extended periods of time, limiting their potential for dispersal by birds or other overland travel. Dispersal therefore likely occurs primarily within drainages, yet somehow lancines maintain high genetic homogeneity across much larger distances than *Juga* and *Fluminicola*, despite apparently similar ecology.

Thus, the present data supports recognition of the Banbury Springs lanx as a distinct genus and species. However, variation within *Fisherola* and *Lanx* seems to be largely ecophenotypic, giving a total of only four extant species in the subfamily Lancinae.

## Systematic descriptions

### Family Lymnaeidae Rafinesque, 1815

#### Idaholanx

Taxon classificationAnimaliaGastropodaLymnaeidae

Clark, Campbell & Lydeard gen. n.

http://zoobank.org/5E7508F1-1AF1-4051-AFD3-E7733DEF094F

##### Type species.

*Idaholanx fresti* Clark, Campbell & Lydeard sp. n.

##### Description.

**Figure 3.**
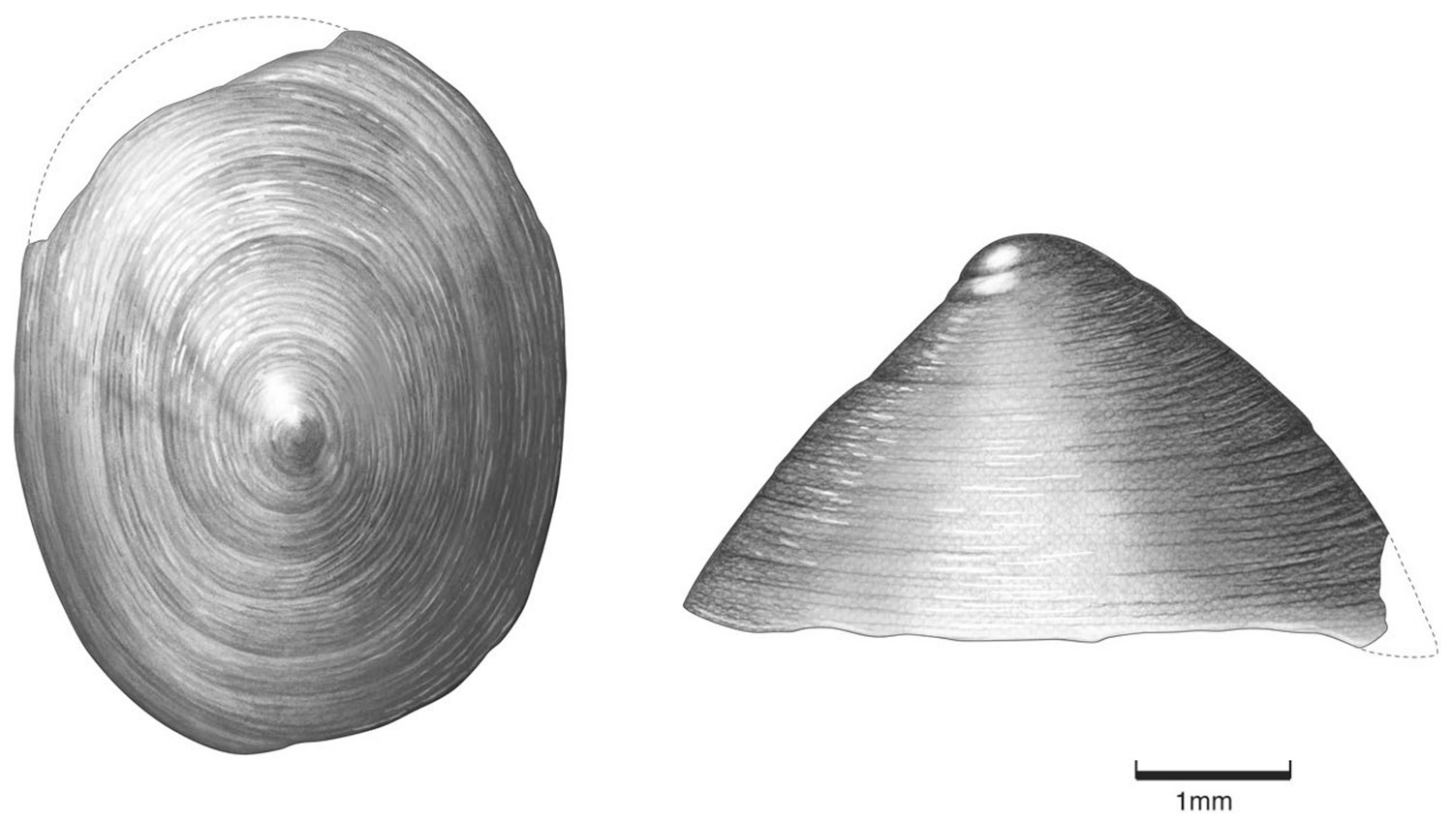
Shell, holotype of *Idaholanx fresti* sp. n.

*Shell* (Figs 3, 5A). Patelliform, 2.0–3.9 mm in height and 4.0–6.7 mm in length and 3.0–5.4 mm in width. Aperture elliptical. Protoconch smooth, apex positioned posteriorly. Teleoconch sculpture of concentric growth lines. Shell pale to dark reddish brown. Internal columellar muscle scar C-shaped.

*Non -genital anatomy*. Columnar muscle C-shaped (Fig. 5B), gap on right side, roughly central. Digestive gland, kidney and lung typical of Lymnaeidae and that seen for *Lanx* and *Fisherola* (Baker 1925, SAC personal observations). Animal colour dark grey to black.

**Figure 4.**
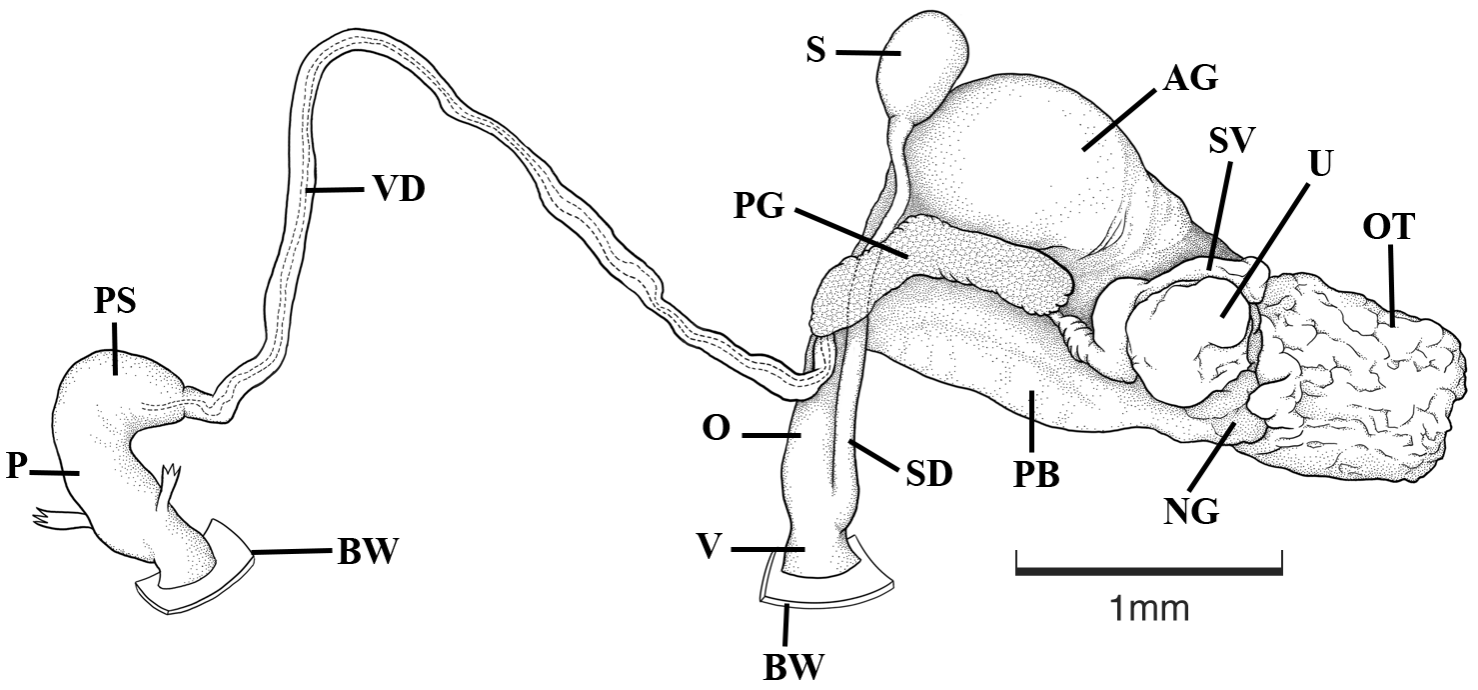
Reproductive anatomy, holotype of *Idaholanx fresti* sp. n. **AG** albumen gland **BW** body wall **NG** nidamental gland **O** oviduct **OT** ovotestis **P** penis **PB** pyriform body **PG** prostate gland **PS** penial sheath **S** spermatheca **SD** spermathecal duct **SV** seminal vesicle **U** uterus **V** vagina **VD** vas deferens.

*Genitalia anatomy* (Fig. 4): The distinction between the praeputium and penial sheath is not clearly defined, the praeputium and the penial sheath are both about half the length of the penial complex. Penis is short and thick. The prostate is elongate and tube like, with the vas deferens entering apically. The uterus is strongly folded, and is surrounded by a large albumen gland. The uterus connects to the proximal part of the oviduct (oviduct I) by a short tubular duct. A roundish nidamental gland joins here. The oviduct widens into the pyriform body which is relatively large, with the anterior portion slightly more swollen than the distal portion. The short oviduct II terminates with a small vagina. The spermatheca is of moderate size and ovate. The spermathecal duct is long and widens at its opening to the vagina.

##### Distribution.

*Idaholanx*, as currently recognised, is known from four isolated cold water springs (Thousand, Banbury, Briggs and Box Canyon Springs) that flow into eastern side of an 8 km section of the Snake River, in Gooding County, Idaho.

##### Remarks.

**Figure 5.**
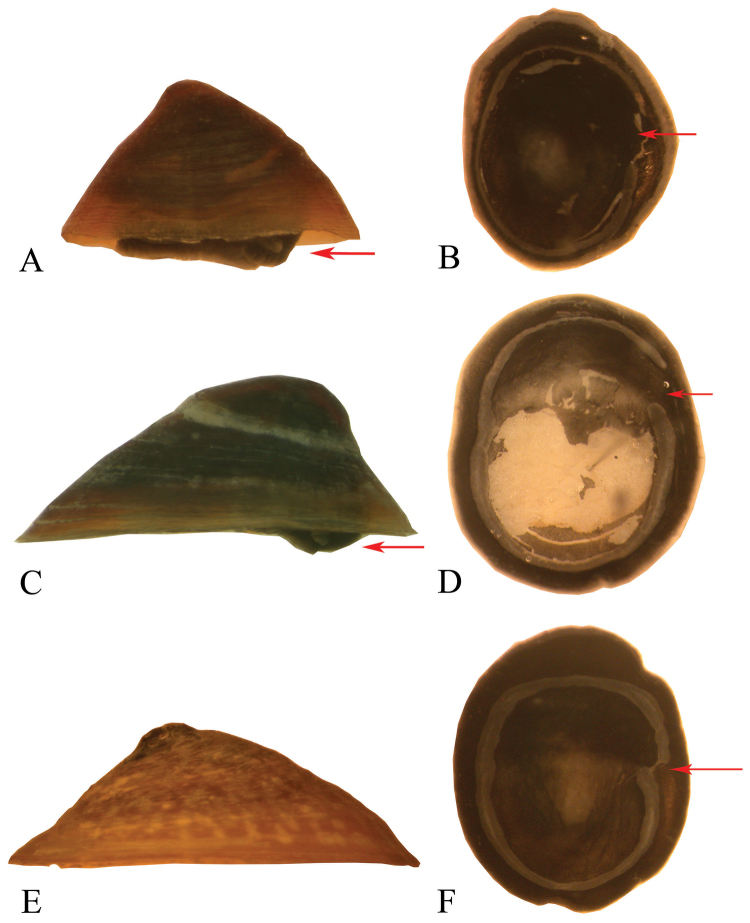
Comparison of shells and animals of *Idaholanx* n. gen., *Fisherola* and *Lanx*. The shells are oriented with the head of the animal facing right, while the whole animals without shells are dorsal views with the head up. *Idaholanx fresti* sp. n. **A** shell **B** whole animal. *Fisherola nuttalli*: **C** shell **D** whole animal. *Lanx patelloides*. **E** shell **F** whole animal. The red arrows indicate the position of the head in **A, C**; the position of the gap in the columella muscle in **B, D** and the narrow connection in **F**. Images not to scale.

*Idaholanx* gen. n. differs from *Fisherola* by having a smaller, taller shell with its apex located towards the middle of the shell and not posteriorly. It differs from *Lanx* by being smaller and taller and having an open C-shaped columellar muscle and not a closed circular columellar muscle (Fig. 5C–F).

##### Etymology.

A combination of Idaho, the only state the genus is known to occur in and *Lanx*, the genus it has been historically referred to and which is currently only known from northern California and southern Oregon.

#### Idaholanx fresti

Taxon classificationAnimaliaGastropodaLymnaeidae

Clark, Campbell & Lydeard sp. n.

http://zoobank.org/9B243DB3-ABD2-40CC-B9A0-BC4DD1778971

##### Type locality.

21–24^th^ runs of the lower outflow of Banbury Springs, Gooding County, Idaho, U.S.A. 42°41'20.5"N, 114°49'18"W, 879m, 4 Sept 2003. Coll: T. Frest & E. Johannes.

##### Type material.

Holotype Field Museum of Natural History (FMNH) 342894 (dissected), paratypes FMNH 342895, DCS, SAC S.26084; FMNH 342896, DCS, SAC S.26085 (shell), 13–15^th^ runs of the lower outflow of Banbury Springs, about middle of spring complex along trail with wooden bridges, 42°41'21"N, 114°49'18"W, 21 Sept 1989; FMNH 342901, lower outflow of Banbury Springs, 42°41'21.8"N, 114°49'19.4"W, 11 Jan 2006; FMNH 342904, SAC S.23967 (shell), lower outflow of Banbury Springs, 42°41'21"N, 114°49'18"W, 6 Aug 2006; FMNH 342897 (shells), SAC S.25699 (shell), lower outflow of Banbury Springs, 42°41'21.8"N, 114°49'18.5"W, 25 May 2016.

##### Additional material examined.

Idaho. *Gooding County*. FMNH 342905 (shells), SAC S.25842 (shell) lower outflow of Box Canyon Spring, about 110m below diversion dam, 42°42'26.5"N, 114°49'02"W, 24 May 2016; FMNH 342898 (shells) lower outflow of Box Canyon Spring, about 160m below diversion dam, 42°42'27"N, 114°49'04"W, Apr 2016; FMNH 342899 (1 dissected), FMNH 342900 (shell) lower outflow of Box Canyon Spring, about 400m below diversion dam, 42°42'27.5"N, 114°49'14.5"W, 11 Jan 2006; FMNH 342902 (1 dissected) outflow of Briggs Spring just below road crossing, 42°40'26.3"N, 114°48'33.4"W, 24 Jan 2006; FMNH 342906 (shells), SAC S.25707 (shell) outflow of Briggs Spring about 15m below diversion dam, 42°40'26.9"N, 114°48'39.2"W, 24 May 2016; FMNH 342903 (1 dissected), outflow of Thousand Springs, 42°44'51.7"N, 114°50'42.3"W, 24 Jan 2006.

##### Description.

Shell and anatomical description as for genus. Holotype 2.8 mm in height, 4.8 mm in length and 3.6 mm in width.

##### Etymology.

Named for the late Dr Terrence J. Frest, for his significant contribution to the knowledge of land and freshwater molluscs of North America, especially of the western states and who was also a colleague and friend.

##### Ecology.

This species is found under and on the sides of stones in cold flowing water in the range of 12.2–16.7 °C. It is not known exactly when egg laying occurs or how many eggs are laided at a time. It could be similar to the closely related species *Fisherola nuttallii* (Haldeman, 1841) which occurs in the Snake River and other major tributaries of, as well as the main stem of the Columbia River. Coutant and Becker (1970) observed *Fisherola nuttallii* laying transparent, suboval gelatinous egg masses containing between 1–12 eggs laid from April to June in the Washington, U.S.A. portion of the Columbia River. They noted that growth rates increased as the availability of food and temperature increased and that the life span was about a year, with adult mortality increasing rapidly after egg laying and after the temperatures increased above 17.3°C.

##### Distribution.

**Figure 6.**
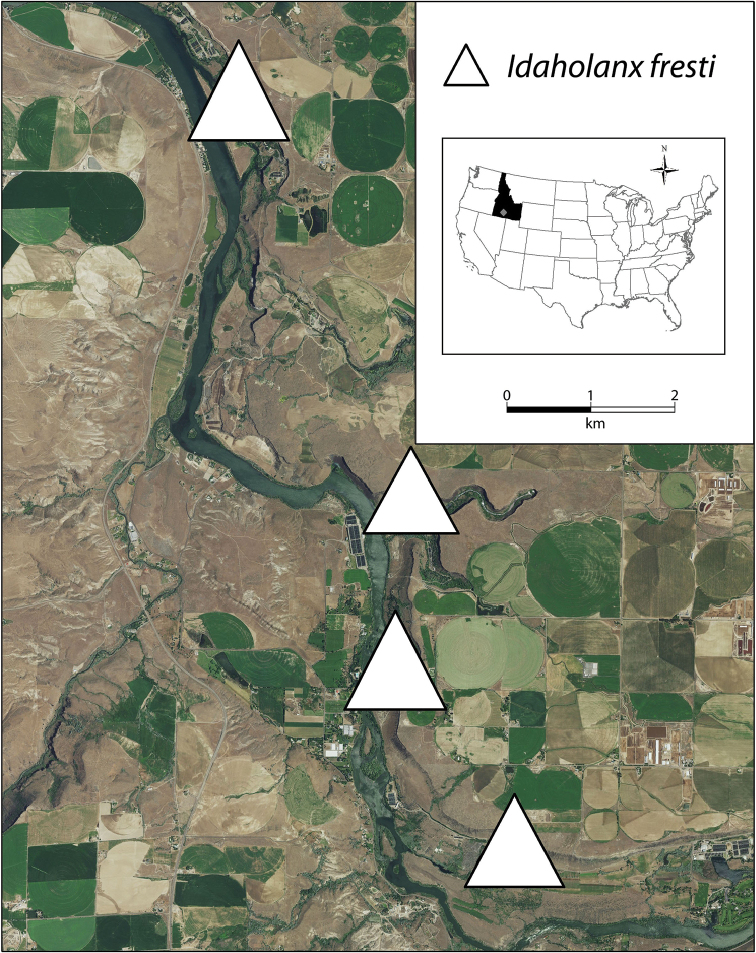
Distribution of *Idaholanx fresti*. Insets show location of Idaho in the US and of the springs in Idaho.

Currently known from four small to large isolated spring complexes along an eight kilometer stretch of the Snake River in Gooding County, Idaho (Fig. 6).

##### Conservation status.

Listed as endangered under the U.S. Endangered Species Act of 1973, under the name Banbury Springs lanx, *Lanx* sp.

## Supplementary Material

XML Treatment for Idaholanx

XML Treatment for Idaholanx fresti

## References

[B1] Albrecht C Kuhn K Streit B (2007) A molecular phylogeny of Planorboidea (Gastropoda, Pulmonata): insights from enhanced taxon sampling. Zoologica Scripta 36: 27–39. https://doi.org/10.1111/j.1463-6409.2006.00258.x

[B2] Albrecht C Wilke T Kuhn K Streit B (2004) Convergent evolution of shell shape in freshwater limpets: the African genus *Burnupia*. Zoological Journal of the Linnean Society 140: 577–586. https://doi.org/10.1111/j.1096-3642.2003.00108.x

[B3] Baker HB (1925) Anatomy of *Lanx*, a limpet-like lymnaeid mollusk. Proceedings of the California Academy of Sciences 14: 143–169.

[B4] Bargues MD Artigas P Dillon RT Mas-Coma S (2009) Molecular characterization of *Lymnaea humilis* (= *L. modicella*), a major fascioliasis vector in North America, and evaluation of the usefulness of nuclear rDNA and mtDNA markers for Lymnaeidae. Unpublished data on GenBank.

[B5] Bargues MD Artigas P Khoubbane M Flores R Glöer P Rojas-García R Ashrafi K Falkner G Mas-Coma S (2011a) *Lymnaea schirazensis*, an overlooked snail distorting fascioliasis data: genotype, phenotype, ecology, worldwide spread, susceptibility, applicability. PLoS ONE 6(9): e24567. http://dx.doi.org/10.1371/journal.pone.0024567 10.1371/journal.pone.0024567 PMC3183092 21980347

[B6] Bargues MD Artigas P Khoubbane M Mas-Coma S (2011b) DNA sequence characterisation and phylogeography of *Lymnaea cousini* and related species, vectors of fascioliasis in northern Andean countries, with description of *L. meridensis* n. sp. (Gastropoda: Lymnaeidae). Parasites & Vectors 4(July): 132 http://dx.doi.org/10.1186/1756-3305-4-132 21749718 10.1186/1756-3305-4-132 PMC3168421

[B7] Bargues MD Artigas P Mera y Sierra RL Pointier JP Mas-Coma S (2007) Characterisation of *Lymnaea cubensis*, *L. viatrix* and *L. neotropica* n. sp., the main vectors of *Fasciola hepatica* in Latin America, by analysis of their ribosomal and mitochondrial DNA. Annals of Tropical Medicine and Parasitology 101(7): 621–641. https://doi.org/10.1179/136485907X229077 17877881 10.1179/136485907X229077

[B8] Bargues MD Mangold AJ Munoz-Antoli C Pointier JP Mas-Coma S (1997) SSU rDNA characterization of lymnaeid snails transmitting human fascioliasis in South and Central America. Journal of Parasitology 83(6): 1086–1092. https://doi.org/10.2307/3284367 9406784

[B9] Bargues MD Mas-Coma S (1997) Phylogenetic analysis of lymnaeid snails based on 18S rDNA sequences. Molecular Biology and Evolution 14(5): 569–577. https://doi.org/10.1093/oxfordjournals.molbev.a025794 9159934 10.1093/oxfordjournals.molbev.a025794

[B10] Bargues MD Mera y Sierra RL Artigas P Mas-Coma S (2012) DNA multigene sequencing of topotypic specimens of the fascioliasis vector *Lymnaea diaphana* and phylogenetic analysis of the genus *Pectinidens* (Gastropoda). Memórias do Instituto Oswaldo Cruz 107(1): 111–124. http://dx.doi.org/10.1590/S0074-02762012000100016 22310544 10.1590/s0074-02762012000100016

[B11] Basch PF (1963) Environmentally influenced shell distortion in a fresh-water limpet. Ecology 44(1): 193–194. https://doi.org/10.2307/1933204

[B12] Bolotov IN Aksenova OV Bespalaya YV Gofarov MY Kondakov AV Paltser IS Stefansson A Travina OV Vinarski MV (2016) Origin of a divergent mtDNA lineage of a freshwater snail species, *Radix balthica*, in Iceland: cryptic glacial refugia or a postglacial founder event? Hydrobiologia 787(1):73–98. https://doi.org/10.1007/s10750-016-2946-9

[B13] Burch JB (1982) Freshwater snails (Mollusca: Gastropoda) of North America. United States Environmental Protection Agency, Cincinnati, vi + 294 pp.

[B14] Burch JB Tottenham JL (1980) North American freshwater snails; Species list, ranges and illustrations. Walkerana 1(3): 81–215.

[B15] Campbell DC Clark SA Johannes EJ Lydeard C Frest TJ (2016) Molecular phylogenetics of the freshwater gastropod genus *Juga* (Cerithioidea: Semisulcospiridae). Biochemical Systematics and Ecology 65: 158–170. https://doi.org/10.1016/j.bse.2016.01.004

[B16] Campbell DC Serb JM Buhay JE Roe KJ Minton RL Lydeard C (2005) Phylogeny of North American amblemines (Bivalvia, Unionoida): prodigious polyphyly proves pervasive across genera. Invertebrate Biology 124(2): 131–164. https://doi.org/10.1111/j.1744-7410.2005.00015.x

[B17] Carpenter JM Wheeler W (1999) Towards simultaneous analysis of morphological and molecular data in Hymenoptera. Zoologica Scripta 28(1-2): 251–260. https://doi.org/10.1046/j.1463-6409.1999.00009.x

[B18] Clessin S (1882) Die familie der Ancylinen. Systematisches Conchylien-Cabinet 1(6): 1–80, plates 1–9.

[B19] Correa AC Escobar JS Noya O Velásquez LE González-Ramírez C Hurtrez-Boussès S Pointier J-P (2011) Morphological and molecular characterization of Neotropic Lymnaeidae (Gastropoda: Lymnaeoidea), vectors of fasciolosis. Infection, Genetics and Evolution 11(8): 1978–1988. http://dx.doi.org/10.1016/j.meegid.2011.09.003 10.1016/j.meegid.2011.09.003 21968212

[B20] Coutant CC Becker CD (1970) Growth of the Columbia River Limpet, *Fisherola nuttalli* (Haldeman), in normal and reactor-warmed water. BNWL-1537, Pacific Northwest Laboratory, Richland, Washington, 14 pp https://doi.org/10.2172/4077701

[B21] Dayrat B Conrad M Balayan S White TR Albrecht C Golding R Gomes SR Harasewych MG De Frias Martins AM (2011) Phylogenetic relationships and evolution of pulmonate gastropods (Mollusca): new insights from increased taxon sampling. Molecular Phylogenetics and Evolution 59(2): 425–437. http://dx.doi.org/10.1016/j.ympev.2011.02.014 21352933 10.1016/j.ympev.2011.02.014

[B22] DeJong RJ Morgan JAT Paraense WL Pointier J-P Amarista M Ayeh-Kumi PFK Babiker A Barbosa CS Bremond P Canese AP de Souza CP Dominguez C File S Gutierrez A Incani RN Kawano T Kazibwe F Kpikpi J Lwambo NJS Mimpfoundi R Njiokou F Poda JN Sene M Velasquez LE Yong M Adema CM Hofkin BV Mkoji GM Loker ES (2001) Evolutionary relationships and biogeography of *Biomphalaria* (Gastropoda: Planorbidae) with implications regarding its role as host of the human bloodfluke, *Schistosoma mansoni*. Molecular Biology and Evolution 18: 2225–2239. https://doi.org/10.1093/oxfordjournals.molbev.a003769 11719572 10.1093/oxfordjournals.molbev.a003769

[B23] Denny MW (2000) Limits to optimization: fluid dynamics, adhesive strength and the evolution of shape in limpet shells. Journal of Experimental Biology 203: 2603–2622. 10934003 10.1242/jeb.203.17.2603

[B24] Dewaard JR Telfer A Young MR (2015) Barcoding Canada Data Release. Data on GenBank.

[B25] Dillon RT Jr McCullough TE Earnhardt CE (2005) Estimates of natural allosperm storage capacity and self-fertilization rate in the hermaphroditic freshwater pulmonate snail, *Physa acuta*. Invertebrate Reproduction and Development 47(2): 111–115. https://doi.org/10.1080/07924259.2005.9652151

[B26] Dowton M Austin AD (2002) Increased congruence does not necessarily indicate increased phylogenetic accuracy — the behavior of the incongruence length difference test in mixed model analyses. Systematic Biology 51: 19–31. https://doi.org/10.1080/106351502753475853 11943090 10.1080/106351502753475853

[B27] Duffy T Kleiman F Pietrokovsky S Issia L Schijman AG Wisnivesky-Colli C (2009) Real-time PCR strategy for rapid discrimination among main lymnaeid species from Argentina. Acta Tropica 109(1): 1–4. https://doi.org/10.1016/j.actatropica.2008.08.003 18983808 10.1016/j.actatropica.2008.08.003

[B28] Feldmeyer B Greshake B Funke E Ebersberger I Pfenninger M (2015) Positive selection in development and growth rate regulation genes involved in species divergence of the genus *Radix*. BMC Evolutionary Biology 15: 164 https://doi.org/10.1186/s12862-015-0434-x 26281847 10.1186/s12862-015-0434-x PMC4539673

[B29] Feldmeyer B Hoffmeier K Pfenninger M (2010) The complete mitochondrial genome of *Radix balthica* (Pulmonata, Basommatophora), obtained by low coverage shot gun next generation sequencing. Molecular Phylogenetics and Evolution 57(3): 1329–1333. http://dx.doi.org/10.1016/j.ympev.2010.09.012 20875865 10.1016/j.ympev.2010.09.012

[B30] Folmer O Hoeh WR Black MB Vrijenhoek RL (1994) DNA primers for amplification of mitochondrial cytochrome C oxidase subunit I from metazoan invertebrates. Molecular Marine Biology and Biotechnology 3: 294–299. 7881515

[B31] Giribet G Carranza S Baguñà J Riutort M Ribera C (1996) First molecular evidence for the existence of a Tardigrada-Arthropoda clade. Molecular Biology and Evolution 13: 76–84. https://doi.org/10.1093/oxfordjournals.molbev.a025573 8583909 10.1093/oxfordjournals.molbev.a025573

[B32] Goloboff P Farris J Nixon K (2008) TNT, a free program for phylogenetic analysis. Cladistics 24: 774–786. https://doi.org/10.1111/j.1096-0031.2008.00217.x

[B33] Gordy MA Kish L Tarrabain M Hanington PC (2016) A comprehensive survey of larval digenean trematodes and their snail hosts in central Alberta, Canada. Parasitology Research 115: 3867–3880. https://doi.org/10.1007/s00436-016-5152-9 27245072 10.1007/s00436-016-5152-9

[B34] Hall TA (1999) BioEdit: a user-friendly biological sequence alignment editor and analysis program for Windows 95/98/NT. Nucleic Acids Symposium Series 41: 95–98.

[B35] Hannibal H (1912) A synopsis of the Recent and Tertiary freshwater Mollusca of the Californian Province, based upon an ontogenetic classification. Proceedings of the Malacological Society of London 10(2): 112–166, plates 5–6.

[B36] Harzhauser M Mandic O (2008) Neogene lake systems of Central and South-Eastern Europe: Faunal diversity, gradients and interrelations. Palaeogeography, Palaeoclimatology, Palaeoecology 260: 417–434. https://doi.org/10.1016/j.palaeo.2007.12.013

[B37] Hershler R Liu H-P Frest TJ Johannes EJ (2007) Extensive diversification of pebblesnails (Lithoglyphidae: *Fluminicola*) in the upper Sacramento River basin, northwestern USA. Zoological Journal of the Linnean Society 149(3): 371–422. https://doi.org/10.1111/j.1096-3642.2007.00243.x

[B38] Holznagel WE Colgan DJ Lydeard C (2010) Pulmonate phylogeny based on 28S rRNA gene sequences: A framework for discussing habitat transitions and character transformation. Molecular Phylogenetics and Evolution 57(3): 1017–1025. http://dx.doi.org/10.1016/j.ympev.2010.09.021 20920591 10.1016/j.ympev.2010.09.021

[B39] Jorgensen A Kristensen TK Stothard JR (2004) An investigation of the ‘Ancyloplanorbidae’ (Gastropoda, Pulmonata, Hygrophila): preliminary evidence from DNA sequence data. Molecular Phylogenetics and Evolution 32(3): 778–787. https://doi.org/10.1016/j.ympev.2004.02.011 15288055 10.1016/j.ympev.2004.02.011

[B40] Jung Y Morgan JAT Burch JB Gordon M Joyce S Laurson J Light J Meyer-Rochov V Pointier J-P DeJong RJ Mkoji GM Loker ES (Unpublished) A phylogeny of the Basommatophora (Gastropoda: Pulmonata), based on 28S and actin sequences. Unpublished data on GenBank.

[B41] Klussmann-Kolb A Dinapoli A Kuhn K Streit B Albrecht C (2008) From sea to land and beyond - new insights into the evolution of euthyneuran Gastropoda (Mollusca). BMC Evolutionary Biology 8(57): 1–16. https://doi.org/10.1186/1471-2148-8-57 18294406 10.1186/1471-2148-8-57 PMC2287175

[B42] Krebs RA Vlasceanu RN Tevesz MJS (2003) An analysis of diversity in freshwater mussels (Bivalvia: Unionidae) of the Cuyahoga and Rocky River watersheds (Ohio, USA) based on the 16S rRNA gene. Journal of Great Lakes Research 29(2): 307–316. https://doi.org/10.1016/S0380-1330(03)70436-5

[B43] Larkin MA Blackshields G Brown NP Chenna R McGettigan PA McWilliam H Valentin F Wallace IM Wilm A Lopez R Thompson JD Gibson TJ Higgins DG (2007) Clustal W and Clustal X version 2.0. Bioinformatics 23: 2947–2948. https://doi.org/10.1093/bioinformatics/btm404 17846036 10.1093/bioinformatics/btm404

[B44] MacNeil FS (1939) Fresh-water invertebrates and land plants of Cretaceous age from Eureka, Nevada. Journal of Paleontology 13(3): 355–360.

[B45] Marquez FJ (Unpublished) Differentiation of *Lymnaea* subgenus (*Galba*, *Leptolymnaea* [sic], *Lymnaea* s.st., *Radix* and *Stagnicola*) (Basommatophora, Lymnaeidae) in base to small ribosomal DNA helix E10-1 sequence. [A very similar set of sequences appears in Bargues and Mas-Coma 1997]

[B46] McMahon RF (2004) A 15-year study of interannual shell-shape variation in a population of freshwater limpets (Pulmonata: Basommatophora: Ancylidae). American Malacological Bulletin 19(1/2): 101–109.

[B47] McMahon RF Whitehead BE (1987) Environmental induction of shell morphometric variation in the European stream limpet, *Ancylus fluviatilis* (Müller) (Pulmonata: Basommatophora). American Malacological Bulletin 5(1): 105–124.

[B48] Meier-Brook C Bargues MD (2002) *Catascopia*, a new genus for three Nearctic and one Palaearctic stagnicoline species (Gastropoda: Lymnaeidae). Folia Malacologia 10(2): 83–84. https://doi.org/10.12657/folmal.010.008

[B49] Morgan JA DeJong RJ Jung Y Khallaayoune K Kock S Mkoji GM Loker ES (2002) A phylogeny of planorbid snails, with implications for the evolution of *Schistosoma* parasites. Molecular Phylogenetics and Evolution 25(3): 477–488. https://doi.org/10.1016/S1055-7903(02)00280-4 12450752 10.1016/s1055-7903(02)00280-4

[B50] Morrison JPE (1955) Notes on the genera *Lanx* and *Fisherola* (Pulmonata). The Nautilus 68(3): 79–83.

[B51] Njiokou F Bellec C Jarne P Finot L Delay B (1993) Mating system analysis using protein electrophoresis in the self-fertile hermaphrodite species *Bulinus truncatus* (Gastropoda: Planorbidae). Journal of Molluscan Studies 59(2): 125–133. https://doi.org/10.1093/mollus/59.2.125

[B52] Nolan JR Bergthorsson U Adema CM (2014) *Physella acuta*: atypical mitochondrial gene order among panpulmonates (Gastropoda). Journal of Molluscan Studies 80: 388–399. https://doi.org/10.1093/mollus/eyu025 25368439 10.1093/mollus/eyu025 PMC4214460

[B53] Nylander JAA (2004) MrModeltest v2. Program distributed by the author. Evolutionary Biology Centre, Uppsala University https://doi.org/10.1006/mpev.1999.0691

[B54] Park D-S Oh H Lee M Kim M Jung C (2012) Korean Collection for Type Cultures. Data on GenBank.

[B55] Park J-K Ó Foighil D (2000) Sphaeriid and corbiculid clams represent separate heterodont bivalve radiations into freshwater environments. Molecular Phylogenetics and Evolution 14(1): 75–88. 10631043 10.1006/mpev.1999.0691

[B56] Patel S Schell T Eifert C Feldmeyer B Pfenninger M (2015) Characterizing a hybrid zone between a cryptic species pair of freshwater snails. Molecular Ecology 24: 643–655. https://doi.org/10.1111/mec.13049 25533031 10.1111/mec.13049

[B57] Pilsbry HA (1925) The family Lancinae distinguished from the Ancylidae. Nautilus 38(3): 73–75.

[B58] Puslednik L Ponder WF Dowton M Davis AR (2009) Examining the phylogeny of the Australasian Lymnaeidae (Heterobranchia: Pulmonata: Gastropoda) using mitochondrial, nuclear and morphological markers. Molecular Phylogenetics and Evolution 52(3): 643–659. https://doi.org/10.1016/j.ympev.2009.03.033 19362157 10.1016/j.ympev.2009.03.033

[B59] Puurtinen MK Knott E Suonpää S Nissinen K Kaitala V (2007) Predominance of outcrossing in *Lymnaea stagnalis* despite low apparent fitness costs of self-fertilization. Journal of Evolutionary Biology 20: 901–912. https://doi.org/10.1111/j.1420-9101.2007.01312.x 17465901 10.1111/j.1420-9101.2007.01312.x

[B60] Remigio EA (2002) Molecular phylogenetic relationships in the aquatic snail genus *Lymnaea*, the intermediate host of the causative agent of fascioliasis: insights from broader taxon sampling. Parasitological Research 88(7): 687–696. https://doi.org/10.1007/s00436-002-0658-8 10.1007/s00436-002-0658-8 12107463

[B61] Remigio EA Blair D (1997) Molecular systematics of the freshwater snail family Lymnaeidae (Pulmonata: Basommatophora) utilising mitochondrial ribosomal DNA sequences Journal of Molluscan Studies 63(2): 173–185. https://doi.org/10.1093/mollus/63.2.173

[B62] Remigio EA Hebert PD (2003) Testing the utility of partial COI sequences for phylogenetic estimates of gastropod relationships. Molecular Phylogenetics and Evolution 29(3): 641–647. https://doi.org/10.1016/S1055-7903(03)00140-4 14615199 10.1016/s1055-7903(03)00140-4

[B63] Ridgway TM Stewart BA Branch GM (1999) Limited population differentiation in the bearded limpet *Patella barbara* (Gastropoda: Patellidae) along the coast of South Africa. Journal of the Marine Biological Association of the United Kingdom 79(4): 639–651. https://doi.org/10.1017/S0025315498000800

[B64] Ronquist F Teslenko M van der Mark P Ayres D Darling A Höhna S Larget B Liu L Suchard MA Huelsenbeck JP (2011) MrBayes 3.2: Efficient Bayesian phylogenetic inference and model choice across a large model space. Systematic Biology 61(3): 539–542. https://doi.org/10.1093/sysbio/sys029 10.1093/sysbio/sys029 PMC3329765 22357727

[B65] Schilthuizen M Hoekstra RF Gittenberger E (1999) Selective maintenance of a rare haplotype in a land snail hybrid zone. Proceedings of the Royal Society of London, Biological Sciences 266(1434): 2181–2185. https://doi.org/10.1007/s00239-004-0232-3

[B66] Simpson RJ Wilding CS Grahame J (2005) Intron analyses reveal multiple calmodulin copies in *Littorina*. Journal of Molecular Evolution 60(4): 505–512. 15883885 10.1007/s00239-004-0232-3

[B67] Sonnenberg R Nolte AW Tautz D (2007) An evaluation of LSU rDNA D1–D2 sequences for their use in species identification. Frontiers in Zoology 4(6): 12 p. http://dx.doi.org/10.1186/1742-9994-4-6 17306026 10.1186/1742-9994-4-6 PMC1805435

[B68] Stothard JR Bremond P Andriamaro L Loxton NJ Sellin B Sellin E Rollinson D (2000) Molecular characterization of the freshwater snail *Lymnaea natalensis* (Gastropoda: Lymnaeidae) on Madagascar with an observation of an unusual polymorphism in ribosomal small subunit genes. Journal of Zoology 252(3): 303–315. https://doi.org/10.1111/j.1469-7998.2000.tb00625.x

[B69] Swofford DL (1998) PAUP*. Phylogenetic Analysis Using Parsimony (*and other methods). Sinauer Associates, Sunderland, Massachusetts.

[B70] Tanaka MO Duque-Estrada TEM Magalhães CA (2002) Dynamics of the acmaeid limpet *Collisella subrugosa* and vertical distribution of size and abundance along a wave exposure gradient. Journal of Molluscan Studies 68(1): 55–64. https://doi.org/10.1093/mollus/68.1.55

[B71] Tryon GW (1870) A Monograph of the Fresh-water Univalve Mollusca of the United- States. Continuation of Prof. S. S. Haldeman’s work. Philadelphia, 1–238. https://doi.org/10.5962/bhl.title.54506

[B72] U.S. Fish and Wildlife Service (1992) Endangered and threatened wildlife and plants: determinations of endangered or threatened status for five aquatic snails in South Central Idaho. Federal Register 57: 59244–59256.

[B73] U.S. Fish and Wildlife Service (2006) Banbury Springs Lanx (*Lanx* n. sp.) (undescribed) 5-Year Review: Summary and Evaluation. U.S. Fish and Wildlife Service Snake River Fish and Wildlife Office, Boise, Idaho, ii+30+VII pp.

[B74] Vermeij GJ (2016) The limpet form in gastropods: evolution, distribution, and implications for the comparative study of history. Biological Journal of the Linnean Society [Online Early view, not yet assigned to a volume]. https://doi.org/10.1111/bij.12883

[B75] Vinarski MV Schniebs K Glöer P Hundsdoerfer AK (2011) The taxonomic status and phylogenetic relationships of the genus *Aenigmomphiscola* Kruglov and Starobogatov, 1981 (Gastropoda: Pulmonata: Lymnaeidae). Journal of Natural History 45(33-34): 2049–2068. https://doi.org/10.1080/00222933.2011.574800

[B76] Vonnemann V Schrödl M Klussmann-Kolb A Wägele H (2005) Reconstruction of the phylogeny of the Opisthobranchia (Mollusca, Gastropoda) by means of 18S and 28S rDNA sequences Journal of Molluscan Studies 71(2): 113–125. https://doi.org/10.1093/mollus/eyi014

[B77] von Oheimb PV Albrecht C Riedel F Du L Yang J Aldridge DC Bößneck U Zhang H Wilke T (2011) Freshwater biogeography and limnological evolution of the Tibetan Plateau – insights from a plateau-wide distributed gastropod taxon (*Radix* spp.). PLoS ONE 6(10): e26307. https://doi.org/10.1371/journal.pone.0026307 10.1371/journal.pone.0026307 PMC3197626 22028853

[B78] Walker SM Makundi AE Namuba FV Kassuku AA Keyyu J Hoey EM Prodohl P Stothard JR Trudgett A (2008) The distribution of *Fasciola hepatica* and *Fasciola gigantica* within southern Tanzania – constraints associated with the intermediate host. Parasitology 135(4): 495–503. https://doi.org/10.1017/s0031182007004076 18205983 10.1017/S0031182007004076

[B79] Walter HJ (1969) Illustrated biomorphology of the ‘*angulata*’ lake form of the basommatophoran snail *Lymnaea catascopium* Say. Malacological Review 2: 1–102.

[B80] Walther AC Taehwan L Burch JB Ó Foighil D (2006a) Confirmation that the North American ancylid *Ferrissia fragilis* (Tryon, 1863) is a cryptic invader of European and East Asian freshwater ecosystems. Journal of Molluscan Studies 72(3): 318–321. https://doi.org/10.1093/mollus/eyl009

[B81] Walther AC Taehwan L Burch JB Ó Foighil D (2006b) *E Pluribus Unum*: A phylogenetic and phylogeographic reassessment of *Laevapex* (Pulmonata: Ancylidae), a North American genus of freshwater limpets. Molecular Phylogenetics and Evolution 40(2): 501–516. https://doi.org/10.1016/j.ympev.2006.03.019 16678447 10.1016/j.ympev.2006.03.019

